# A Bio-Inspired Comprehensive Learning Strategy-Enhanced Parrot Optimizer: Performance Evaluation and Application to Reservoir Production Optimization

**DOI:** 10.3390/biomimetics11020135

**Published:** 2026-02-12

**Authors:** Boyang Yu, Yizhong Zhang

**Affiliations:** Petroleum Engineering School, Yangtze University, Wuhan 430100, China; 2023710393@yangtzeu.edu.cn

**Keywords:** parrot optimizer, comprehensive learning strategy, metaheuristic optimization, reservoir production optimization, bio-inspired algorithm, global optimization

## Abstract

The efficacy of swarm intelligence algorithms in navigating high-dimensional, non-convex landscapes depends on the dynamic balance between global exploration and local exploitation. Drawing inspiration from the intricate social dynamics of *Pyrrhura molinae*, this study proposes a novel bio-inspired metaheuristic, the Comprehensive Learning Parrot Optimizer (CL-PO). While the original Parrot Optimizer (PO) simulates collective foraging and communication, it often suffers from population homogenization and entrapment in local optima due to its reliance on single-source social learning. To address these limitations, CL-PO incorporates a dimension-wise multi-exemplar social learning mechanism analogous to the cross-individual knowledge transfer observed in avian colonies. This strategy enables stagnant individuals to reconstruct their search trajectories by learning from multiple superior peers, thereby sustaining population diversity and facilitating adaptive exploration. Rigorous benchmarking on 29 test functions from the CEC 2017 suite reveals that CL-PO achieves statistically superior performance compared to nine state-of-the-art algorithms, securing a top-tier average Friedman rank of 1.28. Furthermore, the practical utility of CL-PO is substantiated through a complex reservoir production optimization task using the Egg benchmark model, where it consistently maximizes the net present value (NPV), reaching $9.625\times 10^{8}$ USD. These findings demonstrate that CL-PO is a powerful and reliable solver for addressing large-scale engineering optimization problems under complex constraints.

## 1. Introduction

Optimization serves as a cornerstone of modern scientific and engineering research, underpinning critical advancements in fields ranging from artificial intelligence and machine learning to logistics and sustainable energy systems [1,2]. Its principal objective—to systematically identify optimal solutions within vast alternatives under defined constraints—makes it indispensable for enhancing efficiency, performance, and economic viability [3,4]. In practice, however, real-world optimization problems often present formidable computational challenges [5]. Many are formulated within high-dimensional, non-convex, and discontinuous search spaces, characterized by numerous local optima and computationally expensive objective evaluations. These intricacies often render conventional analytical or gradient-based approaches inadequate for a broad spectrum of practical applications [6,7].

Classical optimization methodologies, such as gradient-based techniques and linear programming, are theoretically rigorous and computationally efficient within well-defined domains [8]. Gradient descent excels in locating optima within smooth, convex landscapes, while the simplex method remains a robust standard for linear programming [9]. However, these methods encounter fundamental obstacles when confronted with complex real-world landscapes. Their reliance on derivative information or linearity assumptions becomes a critical weakness in non-convex, multimodal, or discontinuous spaces, where they are prone to entrapment in suboptimal local solutions [10]. Furthermore, their efficacy deteriorates sharply with increasing dimensionality—the “curse of dimensionality”—making the exponential expansion of the search space computationally intractable for traditional approaches. This mismatch between methodological assumptions and the complexity of real-world problems necessitates more robust and adaptable solution strategies [11,12].

To address these limitations, metaheuristic algorithms have emerged as a powerful and versatile class of optimization strategies [13]. Unlike classical methods, metaheuristics navigate complex landscapes without relying on restrictive mathematical properties like gradient information or convexity. They operate based on high-level principles, often inspired by natural phenomena, social behaviors, or physical processes [14]. In recent years, a plethora of innovative metaheuristics have been proposed to tackle increasingly intricate optimization tasks, including the Colony Predation Algorithm (CPA) [15], Moss Growth Optimization (MGO) [16], Educational Competition Optimizer (ECO) [17], and the Beaver Behavior Optimizer (BBO) [18]. These algorithms have demonstrated exceptional performance in diverse domains, from medical image processing and PV parameter identification to complex industrial control systems [19,20,21]. Their core strength lies in robust global search capabilities and a flexible framework that balances exploration (diversification) and exploitation (intensification) [22]. This characteristic enables them to effectively escape local optima and identify near-optimal solutions for problems that are otherwise intractable, positioning metaheuristics as essential tools in modern optimization.

Given the considerable diversity within the metaheuristic paradigm, these algorithms are commonly classified into two principal lineages based on their foundational inspirations: evolutionary algorithms (EAs) and swarm intelligence (SI) algorithms. Evolutionary algorithms are grounded in the principles of biological evolution, primarily employing mechanisms such as selection, crossover, and mutation to evolve a population of candidate solutions over successive generations, mimicking natural selection. Prominent representatives include the Genetic Algorithm (GA) [23] and Differential Evolution (DE) [24]. In contrast, swarm intelligence algorithms derive their methodology from the collective, decentralized behaviors observed in social animal colonies, such as flocks or ant colonies. These algorithms guide a population of agents through the search space using simple rules that model cooperation and information sharing. Notable examples are Particle Swarm Optimization (PSO) [25], which simulates the social dynamics of bird flocking, and Ant Colony Optimization (ACO) [26], which abstracts the foraging behavior of ants using pheromone trails. This taxonomic distinction provides a structured framework for understanding the dominant design philosophies in metaheuristic optimization and underscores the breadth of natural and social metaphors harnessed for problem-solving.

The proliferation of metaheuristics is fundamentally supported by the No Free Lunch (NFL) theorem [27], which posits that no single algorithm can outperform all others across all possible optimization problems. This theoretical insight suggests that superior performance in one domain is inevitably compensated for by inferior performance in another, providing a strong impetus for the development of specialized or enhanced methodologies. While metaheuristics offer powerful general-purpose strategies, they often face challenges such as premature convergence, sensitivity to parameters, and the delicate trade-off between exploration and exploitation. Consequently, ongoing research remains essential to refine their convergence properties and extend their applicability to complex, domain-specific tasks.

This work focuses on the recently proposed Parrot Optimizer (PO) [19], a swarm intelligence metaheuristic inspired by the social dynamics of *Pyrrhura molinae* parrots. The motivation for choosing PO as the baseline for this research is threefold. First, PO’s “four behavioral rules” offer a unique and flexible framework that naturally mimics social dynamics, providing a versatile foundation for optimization. Second, our preliminary analysis revealed a specific “architectural vulnerability”: its reliance on single-source social learning makes it particularly prone to diversity loss in the late stages of search, presenting a clear opportunity for improvement. Third, the stochastic selection of behaviors in PO makes it an ideal candidate for testing the effectiveness of multi-source learning strategies across different update patterns. Since its inception, PO has garnered significant attention and has been successfully applied to various domains, including control engineering, feature selection, and energy system dispatch [19]. Recent variants have also explored hybridizations with other mechanisms to further enhance its search capability suitable for specific landscapes.

However, a critical analysis of the original PO reveals a unified yet restrictive learning paradigm: position updates are predominantly governed by attraction toward either the global best solution or the population mean. This single-source guidance constitutes a significant architectural vulnerability; as iterations progress, the population rapidly loses diversity and converges toward a limited region. This limitation severely hinders the algorithm’s ability to maintain parallel exploration in multimodal landscapes, rendering it susceptible to stagnation in local optima.

To address these shortcomings, this paper introduces the Comprehensive Learning Parrot Optimizer (CL-PO). The central innovation is the conditional integration of a comprehensive learning strategy (CLS) for stagnant individuals. Rather than learning holistically from a single attractor, a stagnant agent constructs a composite guiding vector by learning dimension by dimension from the historical best positions of various superior peers. This multi-source learning mechanism dramatically diversifies search trajectories, effectively breaking the pull of local optima. The contributions of this study are threefold:

- We propose CL-PO, which seamlessly integrates CLS with the original PO behaviors to provide a targeted escape mechanism;
- The performance is rigorously validated against the original PO and state-of-the-art metaheuristics on comprehensive benchmark functions, with statistical tests confirming superior solution accuracy and convergence reliability;
- We demonstrate the practical utility of CL-PO by applying it to the high-stakes reservoir production optimization problem, consistently achieving higher net present value (NPV) and greater reliability than its peers.

The remainder of this paper is organized as follows. Section 2 reviews the original PO and its specific limitations. Section 3 details the proposed CL-PO and its integration mechanics. Section 4 presents the experimental setup and performance analysis on benchmark functions. Section 5 demonstrates the application of CL-PO to reservoir production optimization. Finally, Section 6 summarizes the key findings and suggests future research directions.

## 2. Original Parrot Optimizer

The Parrot Optimizer (PO), proposed by Lian et al. in 2024 [19], is a population-based metaheuristic algorithm inspired by the social behaviors of domesticated *Pyrrhura molinae* parrots. Its core optimization principle involves modeling a flock of *N* parrots, where each individual’s position in a *D*-dimensional space represents a candidate solution. Unlike algorithms with distinct exploration and exploitation phases, PO employs a stochastic update mechanism where each parrot randomly selects one of four biological behaviors—Foraging, Staying, Communicating, and Fear of Strangers—in each iteration to update its position. This design aims to enhance population diversity and mitigate the risk of premature convergence.

The algorithm translates from its biological inspiration to a formal mathematical model comprising initialization, four behavior-specific update equations, and an iterative optimization loop.

### 2.1. Mathematical Model of PO

1. Population initialization: The algorithm commences by randomly generating the initial position for each parrot (candidate solution) within the predefined search space bounds. This establishes a diverse starting population for the iterative search process, which is calculated as follows:

(1) $$X_{i}^{0}=lb+\mathrm{rand}\left(0,1\right)\cdot \left(ub-lb\right).$$

where $X_{i}^{0}$ is the initial position of the *i*-th parrot; lb and ub are the lower and upper bounds of the search space; and rand(0,1) is a uniform random number in [0,1].

2. Foraging behavior: Inspired by parrots foraging in groups near food sources and their owner, this behavior guides an individual toward promising areas. The update combines a Lévy flight directed by the difference from the global best position ($X_{best}$, representing the owner/food location) and a social component influenced by the flock’s mean position ($X_{mean}$). The positional movement is dictated by

(2) $$X_{i}^{t+1}=\left(X_{i}^{t}-X_{best}\right)\cdot \mathrm{Levy}\left(D\right)+\mathrm{rand}\left(0,1\right)\cdot {\left(1-\frac{t}{Max_{iter}}\right)}^{\frac{2t}{Max_{iter}}}\cdot X_{mean}^{t}.$$

where $X_{i}^{t}$ is the current position; $X_{best}$ is the best-found position so far; Levy(D) denotes a Lévy flight random step; and $X_{mean}^{t}$ is the population’s mean position at iteration *t*. The Lévy flight, a type of random walk pattern commonly observed in natural foraging behaviors, introduces large-scale exploratory jumps. It is calculated as $\mathrm{Levy}\left(D\right)=\frac{\mu \cdot \sigma }{{\left|v\right|}^{1/\gamma }}$, where μ and *v* are random values drawn from a standard normal distribution, γ=1.5, and σ is a scaling parameter involving the Gamma function.

3. Staying behavior: Modeling a parrot perching randomly on its owner, this behavior adds a random step based on the owner’s position ($X_{best}$) and a small stochastic vector. It represents localized, random movement around a trusted point, formulated as

(3) $$X_{i}^{t+1}=X_{i}^{t}+X_{best}\cdot \mathrm{Levy}\left(D\right)+\mathrm{rand}\left(0,1\right)\cdot \mathrm{ones}\left(1,D\right).$$

where ones(1,D) is a *D*-dimensional vector with all elements equal to 1, introducing uniform random perturbation.

4. Communicating behavior: This behavior simulates two equally probable social actions: flying to the flock’s center to communicate or moving randomly while communicating. Both sub-behaviors apply incremental position updates. The process is modeled as

(4) $$X_{i}^{t+1}=\left\{\begin{array}{cc} X_{i}^{t}+0.2\cdot \mathrm{rand}\left(0,1\right)\cdot \left(1-\frac{t}{Max_{iter}}\right)\cdot \left(X_{i}^{t}-X_{mean}^{t}\right), & P\leq 0.5;\\ X_{i}^{t}+0.2\cdot \mathrm{rand}\left(0,1\right)\cdot \exp\left(-\frac{t}{\mathrm{rand}\left(0,1\right)\cdot Max_{iter}}\right)\cdot X_{mean}^{t}, & P>0.5. \end{array}\right.$$

where *P* is a uniform random number in [0,1] determining which sub-behavior is executed.

5. Fear of strangers’ behavior: Driven by a natural aversion to unfamiliar individuals, this behavior models a parrot fleeing from strangers and seeking safety with its owner ($X_{best}$). The movement is modulated by cosine functions, creating oscillatory attraction towards the owner and repulsion from perceived threats:

(5) $$\begin{array}{cc} X_{i}^{t+1}= & X_{i}^{t}+\mathrm{rand}(0,1)\cdot \cos\left(0.5\pi \cdot \frac{t}{Max_{iter}}\right)\cdot \left(X_{best}-X_{i}^{t}\right)\\ \; & -\cos(\mathrm{rand}\left(0,1\right)\cdot \pi )\cdot {\left(\frac{t}{Max_{iter}}\right)}^{\frac{2}{Max_{iter}}}\cdot \left(X_{i}^{t}-X_{best}\right). \end{array}$$

The first cosine term regulates flight toward the owner, while the second term governs movement away from strangers.

### 2.2. Integrated Iteration

**Figure 1:**
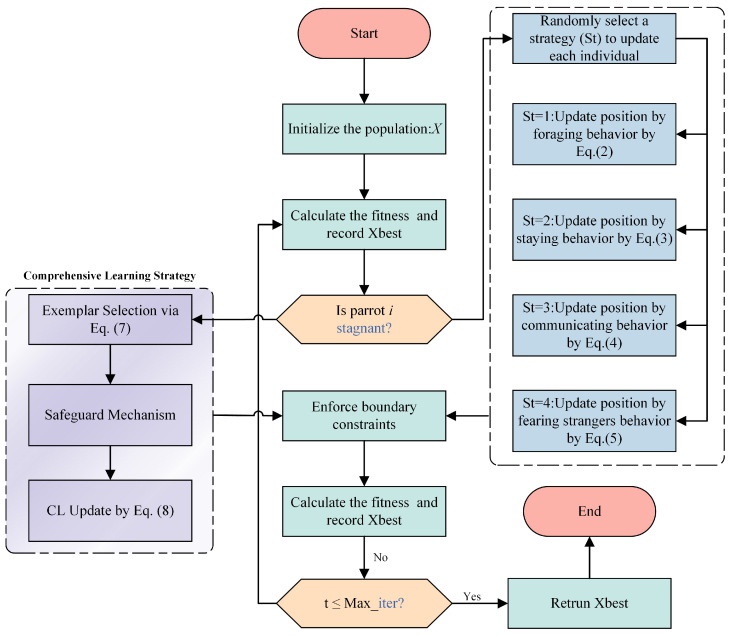
Comparative workflow of the original PO and the proposed CL-PO, highlighting the dual-track mechanism. The blue text represents specific logic components.

The complete PO workflow operates as follows. First, the population is initialized, and the global best position $X_{best}$ is identified. The algorithm then enters the main loop for $T_{max}$ iterations. In each iteration, the mean position $X_{mean}$ of the entire population is computed. Subsequently, for every individual parrot, one of the four behaviors is selected uniformly at random (25% probability each). The parrot’s position is updated according to the corresponding mathematical model. After all individuals are updated, their fitness is evaluated. Each parrot’s personal best and the swarm’s global best ($X_{best}$) are updated if better solutions are found. This process repeats until the termination criterion (maximum iterations) is met, upon which the final $X_{best}$ solution is output. The complete optimization procedure is visually summarized in Figure 1.

### 2.3. Pseudocode of the Original PO

The algorithmic framework of the Parrot Optimizer is formally presented in Algorithm 1. The pseudocode delineates the complete iterative process, from initialization through behavior-based position updates to fitness evaluation and best solution tracking.
**Algorithm 1** Original Parrot Optimizer (PO)1:**Input:** Population size *N*, Maximum iterations Max_iter, Dimension *D*, Boundaries [lb,ub].2:**Initialize** population *X* randomly within [lb,ub] using Equation (1).3:Set Lévy flight parameter γ=1.5.4:Evaluate initial fitness for all individuals.5:Identify global best position $X_{best}$.6:**for** t=1 to Max_iter **do**7:     Calculate population mean position $X_{mean}$ of the flock.8:     **for** i=1 to *N* **do**9:          Randomly select behavior index St∈{1,2,3,4} with equal probability.10:        **if** St=1 **then**11:             Update position $X_{i}$ using **Foraging Behavior** (Equation (2)).12:        **else if** St=2 **then**13:             Update position $X_{i}$ using **Staying Behavior** (Equation (5)).14:        **else if** St=3 **then**15:             Generate random probability P∈[0,1].16:             Update position $X_{i}$ using **Communicating Behavior** (Equation (6)).17:        **else if** St=4 **then**18:             Update position $X_{i}$ using **Fear of Strangers Behavior** (Equation (7)).19:        **end if**20:        Enforce boundary constraints: clip $X_{i}$ to [lb,ub].21:     **end for**22:     Evaluate fitness for all updated individuals.23:     Update global best $X_{best}$ if a better solution is found.24:**end for**25:**Output:** Global best solution $X_{best}$ and its fitness value.

## 3. The Proposed CL-PO Algorithm

This section outlines the core components of the Comprehensive Learning Parrot Optimizer (CL-PO), specifically the integration of the comprehensive learning strategy (CLS) and the augmented algorithmic framework.

### 3.1. Comprehensive Learning Strategy (CLS)

The primary enhancement introduced in this work is the comprehensive learning strategy, which aims to diversify information sources available to individuals during the search process. Instead of relying exclusively on a single global attractor, CLS enables stagnant individuals to selectively inherit information from the dimension-wise best-performing dimensions of multiple peers. This approach synthesizes a composite guiding exemplar that effectively revitalizes localized search trajectories. The strategy comprises three integrated components: adaptive learning probability, dimension-wise exemplar construction, and a safeguard mechanism.

First, an individual-specific learning probability, $P_{c_{i}}$, is assigned to each parrot to regulate the trade-off between self-guided exploitation and peer-informed exploration. This probability is defined as

(6) $$P_{c_{i}}=a+b\times \frac{\exp\left(10\cdot \frac{i-1}{N}\right)-1}{\exp(10)-1},$$

where *a* and *b* are set to 0 and 0.5, respectively, and *i* represents the individual’s rank index.

Second, the learning exemplar vector $f_{i}$ is constructed by determining the target source for each dimension *j* via tournament selection. For each dimension, the algorithm compares the fitness values of the personal best positions of two distinct, randomly selected peers ($k_{1},k_{2}\neq i$):

(7) $$f_{i}\left(j\right)=\left\{\begin{array}{cc} \arg\min_{k\in \{k_{1},k_{2}\}}\mathrm{pfit}\left(k\right), & \mathrm{if}\;\mathrm{rand}<P_{c_{i}};\\ i, & \mathrm{otherwise}. \end{array}\right.$$

Finally, a safeguard mechanism is implemented to prevent complete self-reliance, ensuring that at least one dimension is learned from a peer: if $f_{i}\left(j\right)=i$ for all j∈{1,…,D}, then a randomly chosen dimension $j^{\prime }$ is forced to learn from a superior peer $k_{3}\neq i$.

It is important to differentiate the proposed CLS from the original Comprehensive Learning Particle Swarm Optimizer (CLPSO) [28]. While both approaches leverage the concept of “comprehensive learning,” CL-PO differs significantly in its implementation and target framework. Unlike CLPSO, which applies CL to all particles in every generation, CL-PO uses a “dual-track” mechanism where CLS is only conditionally triggered for individuals that have exceeded a stagnation threshold δ. This maintains the unique biological behaviors of PO while using CLS as a surgical intervention for diversity recovery. Furthermore, the learning exemplar construction in CL-PO is adapted to suit the multi-behavioral update rules of the Parrot flock, which is fundamentally different from the velocity-update mechanism in standard PSO.

### 3.2. Implementation Framework of CL-PO

The proposed CL-PO integrates CLS into the standard PO framework as a conditionally triggered phase based on stagnation detection. A counter, $sc_{i}$, tracks consecutive iterations without improvement for each individual’s personal best. When $sc_{i}$ exceeds a predefined threshold δ, the individual enters the CLS phase. This targeted intervention allows the original PO behaviors to drive exploration under normal conditions, while CLS provides a robust mechanism to escape local optima when progress stalls.

The iterative progression of CL-PO follows a dual-track mechanism. Non-stagnant individuals execute the original PO behavioral updates (Section 2.1). Conversely, for stagnant individuals, the CLS path is activated to construct the learning exemplar $f_{i}$ and perform the position update as

(8) $$X_{i}^{t+1}\left(j\right)=X_{i}^{t}\left(j\right)+\mathrm{rand}\cdot \left(pbest_{f_{i}\left(j\right)}\left(j\right)-X_{i}^{t}\left(j\right)\right).$$

Following this, all new positions undergo evaluation, and fitness records are updated greedily. This dimension-wise, multi-source learning directly counteracts the diversity loss inherent in the original PO, sustaining robust exploration across complex landscapes.

The comprehensive workflow is illustrated in Figure 1, with the formal procedure detailed in Algorithm 2. This dual-track structure preserves the rapid convergence of PO while injecting necessary diversity for multimodal optimization.

### 3.3. Complexity Analysis

The computational complexity of the original PO is O(T·N·D) for *T* iterations. The CLS integration adds a conditional overhead for stagnant individuals: for each such agent, constructing the exemplar vector and updating positions requires O(D). In the worst-case scenario where all individuals are stagnant in every generation, the added complexity is O(T·N·D). Consequently, the overall time complexity of CL-PO remains O(T·N·D), which is asymptotically equivalent to the original PO. This ensures that the global search enhancements are achieved without elevating the order of computational cost.
**Algorithm 2** Comprehensive Learning Parrot Optimizer (CL-PO).1:**Input:** Population size *N*, Max iterations Max_iter, Dimension *D*, Stagnation threshold δ.2:**Initialize** population *X*; Evaluate fitness; Initialize pbest and gbest.3:**Initialize** stagnation counters $sc_{i}=0$ for all individuals.4:Pre-calculate learning probabilities $P_{c_{i}}$ via Equation (8).5:**for** t=1 to Max_iter **do**6:      **for** i=1 to *N* **do**7:            **if** $sc_{i}\geq \delta$ **then**             ▹ Stagnation-triggered CLS Phase8:                 **for** j=1 to *D* **do**9:                      Select exemplar index $f_{i}\left(j\right)$ via tournament selection.10:                **end for**11:                **Safeguard:** Ensure ∃j such that $f_{i}\left(j\right)\neq i$.12:                Update $X_{i}^{new}\left(j\right)=X_{i}\left(j\right)+\mathrm{rand}\cdot \left(pbest_{f_{i}\left(j\right)}\left(j\right)-X_{i}\left(j\right)\right)$.13:            **else**                    ▹ Base Algorithm Phase14:                Randomly select behavior index St∈{1,2,3,4}.15:                Generate candidate $X_{i}^{new}$ using PO behavioral rules.16:            **end if**17:            Enforce boundary constraints.18:            Evaluate fitness $f(X_{i}^{new})$.19:            **if** $f\left(X_{i}^{new}\right)<f\left(pbest_{i}\right)$ **then**20:                 $pbest_{i}=X_{i}^{new}$; $sc_{i}=0$.21:            **else**22:                 $sc_{i}=sc_{i}+1$.23:            **end if**24:      **end for**25:      Update global best $X_{best}$.26:**end for**27:**Output:** Global best solution $X_{best}$.

## 4. Experimental Results and Analysis

This section presents a comprehensive experimental evaluation to assess the performance, robustness, and convergence behavior of the proposed Comprehensive Learning Parrot Optimizer (CL-PO). All experiments were conducted on a consistent computational platform (Intel Core i7-10700K CPU, 16GB RAM, Santa Clara, CA, USA, MATLAB R2021a) to ensure a fair comparison. The algorithm’s performance was rigorously tested on the CEC2017 benchmark suite under standardized conditions: a population size (*N*) of 30, a maximum of $3\times 10^{5}$ function evaluations (MaxFEs), and 30 independent trials for each function to account for stochastic variability. The critical control parameter for CL-PO, the stagnation threshold δ, was set to 2 to ensure a highly responsive escape mechanism. Performance is primarily quantified using the mean (Mean) and standard deviation (Std) of the best-found fitness values, reflecting solution accuracy and algorithmic stability, respectively.

### 4.1. Benchmark Functions and Experimental Setup

The CEC2017 suite comprises 29 challenging real-parameter numerical optimization problems (F1–F29, excluding F2). These functions are categorized into four distinct groups: unimodal functions (F1–F3) for assessing exploitation efficiency; simple multimodal functions (F4–F10) for evaluating basic exploration; hybrid functions (F11–F20) for testing combined search capabilities; and complex composition functions (F21–F29) with numerous local optima. This diverse categorization allows for a multi-faceted examination of an optimizer’s ability to balance global diversification and local intensification. The detailed properties of these functions are summarized in Table 1.

### 4.2. Performance Comparison and Statistical Analysis

To validate the efficacy of the proposed CL-PO, it was compared against its base version, the original Parrot Optimizer (PO) [19], and eight other prominent metaheuristics: Moss Growth Optimization (MGO), Hunger Games Search (HGS) [29], Grey Wolf Optimizer (GWO) [30], Slime Mould Algorithm (SMA) [31], Sine Cosine Algorithm (SCA) [32], Firefly Algorithm (FA) [33], and Bat Algorithm (BA) [34]. This selection encompasses a range of representative algorithms from recent and classical literature. A fair comparison was ensured by employing identical population sizes, maximum iteration limits, and independent run counts for all competitors.

The comprehensive numerical results are summarized in Table 2, which reports the mean and standard deviation of the fitness values for each algorithm across all 29 functions. The table also includes the overall average rank for each algorithm, calculated via the Friedman test, and a pairwise statistical comparison between CL-PO and the original PO. In this comparison, the symbols ‘+’, ‘=’, and ‘−’ denote that CL-PO’s performance is statistically significantly better than, equivalent to, or worse than PO, respectively, based on a non-parametric test at a 5% significance level.

The results from Table 2 reveal that the proposed CL-PO achieves the best overall performance, securing the top rank with an average Friedman rank of 1.28. Among ten competitors, a rank approaching 1.0 demonstrates that the algorithm’s superiority is not limited to a specific type of problem but extends across multimodal, hybrid, and composition functions, proving its robust global search and local exploitation capabilities. It demonstrates statistically significant superiority (‘+’) over the original PO on 27 out of 29 benchmark functions, with equivalent performance (‘=’) on only 2 functions. This decisive outcome highlights the substantial enhancement brought by the integrated strategies. The original PO itself ranks second, followed by HGS and GWO, indicating a strong baseline. Algorithms such as MFO, FA, and SCA exhibit relatively weaker overall rankings on this diverse set. Furthermore, CL-PO frequently attains very low Std values, signifying high solution consistency and robust convergence stability across independent runs.

To rigorously substantiate these findings, the Wilcoxon signed-rank test was performed at a significance level of α=0.05. The resulting *p*-values, consolidated in Table 3, confirm a consistent statistical advantage for CL-PO. Against nearly all competitors, CL-PO demonstrates significant improvement (p<0.05) across the majority of cases. These results confirm that the performance gains are statistically robust and not attributable to random chance.

**Figure 2:**
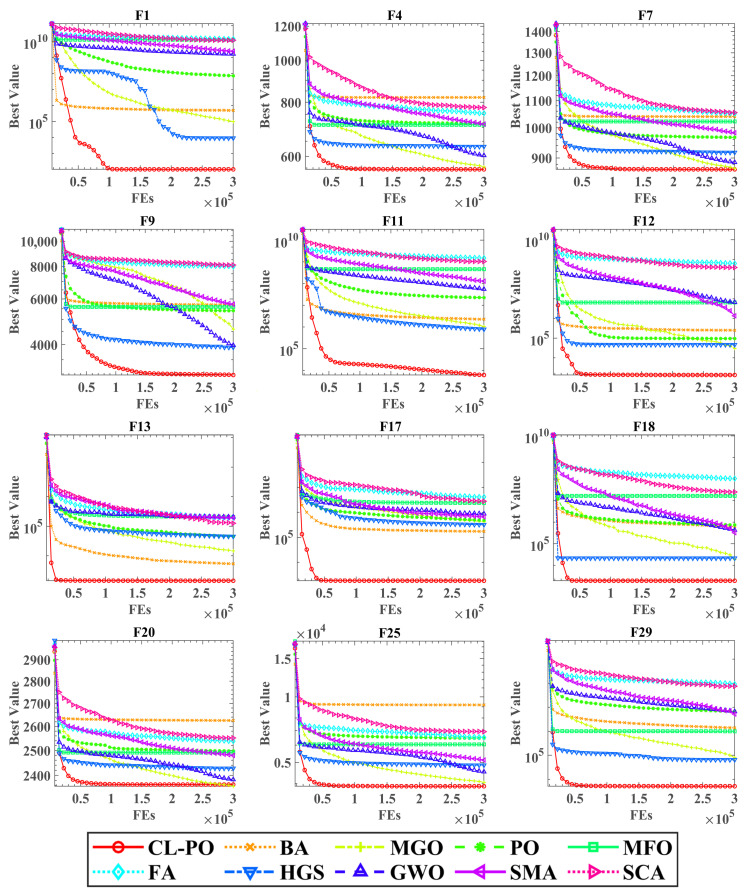
Convergence profiles of CL-PO and competing algorithms on representative benchmark functions.

The convergence behavior, illustrated in Figure 2 for twelve representative functions, provides visual insight into the search dynamics. The figure plots the best fitness value against the number of function evaluations (FEs), using a logarithmic scale for high-magnitude functions. CL-PO is distinctively marked by a red line with circular markers. On the majority of functions, CL-PO exhibits a rapid initial descent, indicating efficient early-stage exploration facilitated by the comprehensive learning strategy. More importantly, it consistently maintains the lowest trajectory throughout the search process, ultimately converging to superior solutions compared to all competitors. This demonstrates the algorithm’s effective balance, where the comprehensive learning strategy limits stagnation and enables escape from local optima in later stages.

In summary, the integrated experimental and statistical analysis confirms that the proposed CL-PO, through its comprehensive learning strategy and multi-exemplar update mechanism, achieves statistically significant and robust performance superiority over its predecessor and a range of established metaheuristics across a diverse benchmark suite.

## 5. Application to Reservoir Production Optimization

Reservoir production optimization represents a formidable engineering challenge, aimed at determining operational strategies that maximize the economic recovery of hydrocarbons, typically quantified by the net present value (NPV) [35]. This problem is inherently high-dimensional and non-deterministic (NP-hard), as the search space is defined by multiple control variables (e.g., well production rates or bottom-hole pressures) across discrete management periods. The resulting complex, non-convex, and computationally intensive objective landscape makes it particularly well-suited for population-based metaheuristics, which navigate such terrains without requiring gradient information.

To evaluate the practical efficacy of CL-PO, we integrated it into a standard reservoir simulation workflow. A high-fidelity synthetic reservoir model was implemented using the Eclipse simulator, serving as the forward model. CL-PO was tasked with optimizing control settings for both production and injection wells, where each candidate solution encoded a complete control schedule for simulation and economic evaluation.

The primary objective is the maximization of NPV, formulated as

(9) $$NPV\left(x,z\right)=\sum_{t=1}^{n}\frac{\Delta t\;(Q_{o,t}\cdot r_{o}-Q_{w,t}\cdot r_{w}-Q_{i,t}\cdot r_{i})}{{\left(1+b\right)}^{p_{t}}},$$

where x and z denote the vectors of control and reservoir state variables, respectively. $Q_{o,t}$, $Q_{w,t}$, and $Q_{i,t}$ represent the average oil production, water production, and water injection rates during period *t*. The coefficients $r_{o}$, $r_{w}$, and $r_{i}$ are the unit prices and costs associated with oil and water handling. The parameter *b* is the annual discount rate, and $p_{t}$ is the cumulative production time.

Physical constraints and operational limits were incorporated using two methods: (1) Box constraints were enforced directly on the control variables (e.g., BHP limits) during the position update phase. (2) Non-linear physical constraints (e.g., maximum water cut or minimum pressure thresholds) were handled via penalty functions within the objective function calculation. If a strategy violates these operational limits during simulation, the NPV is penalized, guiding the optimizer toward feasible regions.

### 5.1. Standard Case: The Egg Benchmark Model

The reservoir model utilized in this study is the well-known Egg model [36], a standardized geological ensemble widely used for waterflooding optimization and closed-loop management research. The model represents a channelized fluvial reservoir system characterized by significant spatial heterogeneity.

**Figure 3:**
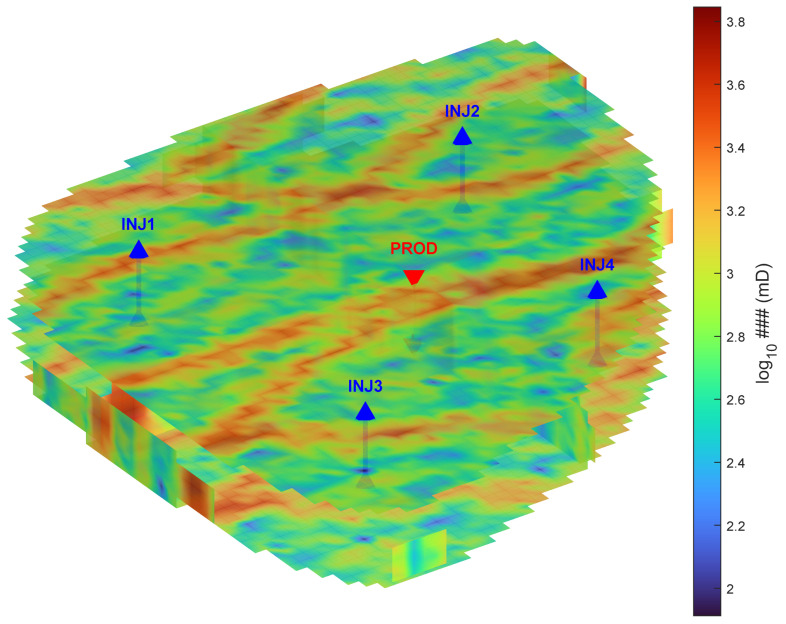
3D permeability distribution of the Egg benchmark model, highlighting channelized heterogeneity and the five-spot well configuration.

Our implementation used a single three-dimensional realization of the Egg model to assess the optimization framework. It should be noted that while this single realization provides a robust baseline for algorithmic comparison, comprehensive validation will require multi-realization studies in future to fully account for geological uncertainty. The model consists of a Cartesian grid (60×60×7) with approximately 18,553 active cells, forming an egg-shaped domain. High-permeability meandering channels are embedded within a low-permeability background, creating preferential flow paths. Production is primarily driven by waterflooding, as the model lacks an aquifer or gas cap. Figure 3 illustrates the 3D permeability field and the five-spot well configuration (four injectors and one central producer).

### 5.2. Experimental Results and Discussion

The proposed CL-PO was benchmarked against the original PO and eight other metaheuristics on the reservoir optimization task. Each algorithm was executed five times to ensure consistency. Performance was evaluated based on the mean NPV, standard deviation (Std), and the best/worst outcomes.

The quantitative results in Table 4 show that CL-PO achieves the highest mean NPV ($9.625\times 10^{8}$ USD) and the lowest standard deviation, indicating enhanced optimization capacity and stability. While MGO and HGS provide competitive results, they exhibit higher variability. Slower-converging algorithms like BA and FA suffer from premature stagnation, resulting in significantly lower economic returns.

**Figure 4:**
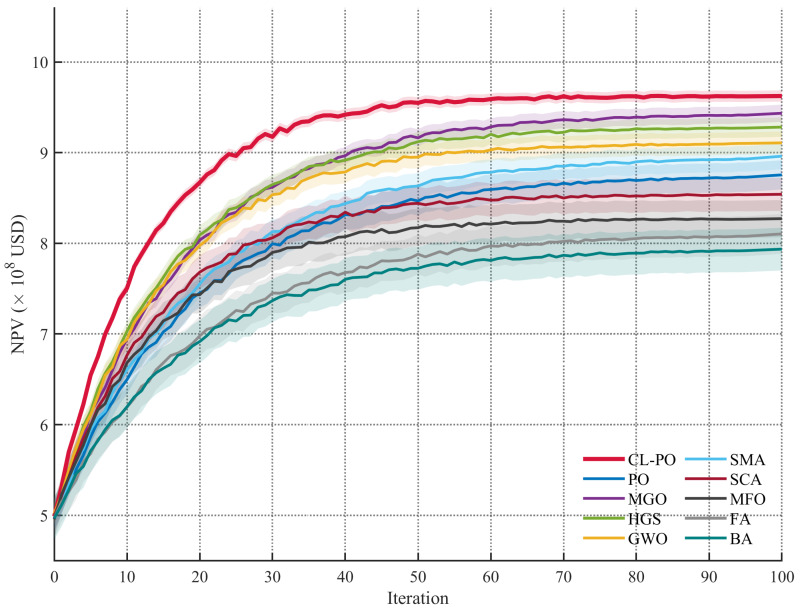
Convergence behavior of different optimizers on the production optimization task.

Convergence trajectories in Figure 4 further confirm CL-PO’s efficiency. Its ascent is more rapid and stable compared to competitors, ultimately reaching a higher plateau. This indicates that the comprehensive learning strategy effectively prevents the algorithm from being trapped in local optima, which is common in high-dimensional reservoir problems. In conclusion, CL-PO demonstrates high reliability and efficacy, making it a promising tool for real-world oilfield development.

## 6. Conclusions

This study introduces the Comprehensive Learning Parrot Optimizer (CL-PO), a robust enhancement of the original Parrot Optimizer (PO) tailored for complex numerical and engineering optimization tasks. The core advancement is the integration of a comprehensive learning strategy (CLS), which enables stagnant individuals to selectively leverage personal best information from multiple peers on a dimension-by-dimension basis. This mechanism directly addresses the architectural limitations of the original PO, specifically its susceptibility to diversity loss and premature convergence in multimodal landscapes.

Rigorous validation on the CEC2017 benchmark suite demonstrates that CL-PO significantly outperforms the base PO and several state-of-the-art metaheuristics. Statistical analyses, including the Friedman and Wilcoxon signed-rank tests, confirm its superior solution accuracy and convergence reliability. Furthermore, the successful application of CL-PO to the reservoir production optimization problem underscores its practical utility in solving high-stakes, high-dimensional engineering challenges, consistently delivering superior net present value with minimal variability.

Future research will explore the extension of CL-PO to constrained and multi-objective optimization domains. Specifically, we aim to investigate the application of CL-PO to large-scale infrastructure planning, such as the spatial allocation of hydrogen electrolyzers, to address interdependent challenges in renewable resource siting [37]. Additionally, integrating reinforcement learning frameworks to dynamically tune the stagnation threshold or behavioral probabilities represents a promising direction for enhancing algorithmic adaptability [38]. In conclusion, the proposed CL-PO offers a powerful, dependable, and computationally efficient tool for the global optimization community.

Notably, the current version of CL-PO primarily addresses box constraints. While it handles some physical limits via penalty functions, further adaptation is required to effectively manage the complex, non-linear geological and engineering constraints typical of real-world oilfield development.

## Figures and Tables

**Table 1:** Summary of CEC2017 benchmark functions.

Function	Title	Category	Optimal Value
F1	Shifted and Rotated Bent Cigar	Unimodal	100
F3	Shifted and Rotated Zakharov	Unimodal	300
F4	Shifted and Rotated Rosenbrock’s	Multimodal	400
F5	Shifted and Rotated Rastrigin’s	Multimodal	500
F6	Shifted and Rotated Expanded Scaffer’s F6	Multimodal	600
F7	Shifted and Rotated Lunacek Bi-Rastrigin	Multimodal	700
F8	Shifted and Rotated Non-Continuous Rastrigin’s	Multimodal	800
F9	Shifted and Rotated Lévy	Multimodal	900
F10	Shifted and Rotated Schwefel’s	Multimodal	1000
F11–F20	Hybrid Functions 1–10	Hybrid	1100–2000
F21–F30	Composition Functions 1–10	Composition	2100–3000

**Table 2:** Results of the CL-PO and other algorithms on CEC2017 benchmark functions. Bold values represent the best performance for each function.

	**F1**		**F3**		**F4**	
**Algo.**	**Avg**	**Std**	**Avg**	**Std**	**Avg**	**Std**
CL-PO	$1.0000\times 10^{2}$	$3.1112\times 10^{-14}$	$3.0086\times 10^{2}$	$2.4798\times 10^{0}$	$4.1923\times 10^{2}$	$2.8546\times 10^{1}$
PO	$7.1889\times 10^{7}$	$1.3016\times 10^{8}$	$5.0589\times 10^{3}$	$3.1669\times 10^{3}$	$5.1584\times 10^{2}$	$2.6588\times 10^{1}$
BA	$5.0096\times 10^{5}$	$3.0718\times 10^{5}$	$3.0011\times 10^{2}$	$1.0045\times 10^{-1}$	$4.9007\times 10^{2}$	$2.5057\times 10^{1}$
MGO	$9.6880\times 10^{4}$	$7.5603\times 10^{4}$	$4.4625\times 10^{4}$	$8.4674\times 10^{3}$	$4.9310\times 10^{2}$	$1.1477\times 10^{1}$
MFO	$1.3096\times 10^{10}$	$7.6028\times 10^{9}$	$1.1258\times 10^{5}$	$6.1804\times 10^{4}$	$1.3204\times 10^{3}$	$8.0947\times 10^{2}$
FA	$1.4504\times 10^{10}$	$1.6232\times 10^{9}$	$5.9888\times 10^{4}$	$1.0009\times 10^{4}$	$1.3856\times 10^{3}$	$1.6022\times 10^{2}$
HGS	$9.1102\times 10^{3}$	$7.0061\times 10^{3}$	$1.2806\times 10^{3}$	$3.8018\times 10^{3}$	$4.8554\times 10^{2}$	$2.8428\times 10^{1}$
GWO	$1.6750\times 10^{9}$	$1.4578\times 10^{9}$	$3.4801\times 10^{4}$	$8.3920\times 10^{3}$	$6.3136\times 10^{2}$	$1.6857\times 10^{2}$
SMA	$2.5090\times 10^{9}$	$1.0756\times 10^{9}$	$3.7499\times 10^{4}$	$6.9297\times 10^{3}$	$6.0451\times 10^{2}$	$4.8424\times 10^{1}$
SCA	$1.2141\times 10^{10}$	$2.1080\times 10^{9}$	$3.6112\times 10^{4}$	$5.2828\times 10^{3}$	$1.4292\times 10^{3}$	$3.0312\times 10^{2}$
	**F5**		**F6**		**F7**	
**Algo.**	**Avg**	**Std**	**Avg**	**Std**	**Avg**	**Std**
CL-PO	$5.5970\times 10^{2}$	$1.4817\times 10^{1}$	$6.0000\times 10^{2}$	$1.1687\times 10^{-3}$	$7.9579\times 10^{2}$	$1.2117\times 10^{1}$
PO	$7.1478\times 10^{2}$	$4.1831\times 10^{1}$	$6.5519\times 10^{2}$	$6.9479\times 10^{0}$	$1.1550\times 10^{3}$	$7.9715\times 10^{1}$
BA	$8.2098\times 10^{2}$	$5.8716\times 10^{1}$	$6.7043\times 10^{2}$	$1.1253\times 10^{1}$	$1.5773\times 10^{3}$	$1.9537\times 10^{2}$
MGO	$5.6704\times 10^{2}$	$1.2930\times 10^{1}$	$6.0000\times 10^{2}$	$7.4570\times 10^{-5}$	$8.0069\times 10^{2}$	$1.3562\times 10^{1}$
MFO	$7.0967\times 10^{2}$	$4.8913\times 10^{1}$	$6.3821\times 10^{2}$	$1.1864\times 10^{1}$	$1.1602\times 10^{3}$	$2.0930\times 10^{2}$
FA	$7.5472\times 10^{2}$	$1.1702\times 10^{1}$	$6.4479\times 10^{2}$	$2.4140\times 10^{0}$	$1.3932\times 10^{3}$	$3.8005\times 10^{1}$
HGS	$6.3203\times 10^{2}$	$2.9265\times 10^{1}$	$6.0189\times 10^{2}$	$1.9860\times 10^{0}$	$9.0486\times 10^{2}$	$5.1462\times 10^{1}$
GWO	$6.0274\times 10^{2}$	$2.4704\times 10^{1}$	$6.0713\times 10^{2}$	$3.0694\times 10^{0}$	$8.7214\times 10^{2}$	$6.2633\times 10^{1}$
SMA	$7.1254\times 10^{2}$	$2.3752\times 10^{1}$	$6.4157\times 10^{2}$	$6.3508\times 10^{0}$	$1.0667\times 10^{3}$	$5.2063\times 10^{1}$
SCA	$7.7826\times 10^{2}$	$1.5945\times 10^{1}$	$6.5109\times 10^{2}$	$5.1143\times 10^{0}$	$1.1286\times 10^{3}$	$4.0904\times 10^{1}$
	**F8**		**F9**		**F10**	
**Algo.**	**Avg**	**Std**	**Avg**	**Std**	**Avg**	**Std**
CL-PO	$8.6491\times 10^{2}$	$1.5023\times 10^{1}$	$9.8563\times 10^{2}$	$7.5731\times 10^{1}$	$3.0562\times 10^{3}$	$3.1813\times 10^{2}$
PO	$9.6739\times 10^{2}$	$2.4844\times 10^{1}$	$5.1500\times 10^{3}$	$7.4870\times 10^{2}$	$5.4044\times 10^{3}$	$5.8777\times 10^{2}$
BA	$1.0401\times 10^{3}$	$5.7327\times 10^{1}$	$1.3728\times 10^{4}$	$3.8682\times 10^{3}$	$5.6920\times 10^{3}$	$6.5201\times 10^{2}$
MGO	$8.6767\times 10^{2}$	$1.1124\times 10^{1}$	$9.4282\times 10^{2}$	$3.5684\times 10^{1}$	$4.5850\times 10^{3}$	$4.5803\times 10^{2}$
MFO	$1.0223\times 10^{3}$	$4.0073\times 10^{1}$	$7.2492\times 10^{3}$	$2.1131\times 10^{3}$	$5.5889\times 10^{3}$	$8.1925\times 10^{2}$
FA	$1.0514\times 10^{3}$	$8.9812\times 10^{0}$	$5.3736\times 10^{3}$	$5.6353\times 10^{2}$	$7.9935\times 10^{3}$	$1.6088\times 10^{2}$
HGS	$9.1767\times 10^{2}$	$2.5278\times 10^{1}$	$3.6808\times 10^{3}$	$8.9033\times 10^{2}$	$3.9048\times 10^{3}$	$4.6024\times 10^{2}$
GWO	$8.8646\times 10^{2}$	$2.5276\times 10^{1}$	$1.9535\times 10^{3}$	$7.0322\times 10^{2}$	$3.9794\times 10^{3}$	$9.5791\times 10^{2}$
SMA	$9.8320\times 10^{2}$	$2.6830\times 10^{1}$	$5.4265\times 10^{3}$	$8.8625\times 10^{2}$	$5.7266\times 10^{3}$	$6.3734\times 10^{2}$
SCA	$1.0549\times 10^{3}$	$1.9389\times 10^{1}$	$5.7202\times 10^{3}$	$9.9690\times 10^{2}$	$8.1009\times 10^{3}$	$2.9810\times 10^{2}$
	**F11**		**F12**		**F13**	
**Algo.**	**Avg**	**Std**	**Avg**	**Std**	**Avg**	**Std**
CL-PO	$1.1265\times 10^{3}$	$1.5435\times 10^{1}$	$6.1185\times 10^{3}$	$8.3345\times 10^{3}$	$1.3428\times 10^{3}$	$1.6465\times 10^{1}$
PO	$1.3085\times 10^{3}$	$6.3082\times 10^{1}$	$2.2513\times 10^{7}$	$2.6434\times 10^{7}$	$9.6185\times 10^{4}$	$5.0975\times 10^{4}$
BA	$1.3067\times 10^{3}$	$6.2183\times 10^{1}$	$2.2739\times 10^{6}$	$1.7459\times 10^{6}$	$2.5010\times 10^{5}$	$9.4646\times 10^{4}$
MGO	$1.1822\times 10^{3}$	$2.5510\times 10^{1}$	$1.0544\times 10^{6}$	$6.8577\times 10^{5}$	$3.0391\times 10^{4}$	$2.4031\times 10^{4}$
MFO	$6.2353\times 10^{3}$	$7.5509\times 10^{3}$	$4.6144\times 10^{8}$	$7.1700\times 10^{8}$	$6.5270\times 10^{6}$	$1.7849\times 10^{7}$
FA	$3.3983\times 10^{3}$	$5.1378\times 10^{2}$	$1.4971\times 10^{9}$	$2.5501\times 10^{8}$	$6.2900\times 10^{8}$	$2.1178\times 10^{8}$
HGS	$1.2121\times 10^{3}$	$3.5176\times 10^{1}$	$7.8373\times 10^{5}$	$6.8638\times 10^{5}$	$4.6680\times 10^{4}$	$2.4720\times 10^{4}$
GWO	$1.9737\times 10^{3}$	$8.8563\times 10^{2}$	$5.5443\times 10^{7}$	$5.4524\times 10^{7}$	$6.7075\times 10^{6}$	$2.2584\times 10^{7}$
SMA	$1.5582\times 10^{3}$	$1.6856\times 10^{2}$	$1.2941\times 10^{8}$	$6.6298\times 10^{7}$	$1.3695\times 10^{6}$	$1.2923\times 10^{6}$
SCA	$2.0911\times 10^{3}$	$2.9733\times 10^{2}$	$1.0440\times 10^{9}$	$2.4882\times 10^{8}$	$3.8987\times 10^{8}$	$1.3685\times 10^{8}$
	**F14**		**F15**		**F16**	
**Algo.**	**Avg**	**Std**	**Avg**	**Std**	**Avg**	**Std**
CL-PO	$1.4323\times 10^{3}$	$1.2862\times 10^{1}$	$1.5214\times 10^{3}$	$1.7066\times 10^{1}$	$2.2078\times 10^{3}$	$1.5370\times 10^{2}$
PO	$4.5127\times 10^{4}$	$2.8464\times 10^{4}$	$7.7866\times 10^{4}$	$5.3192\times 10^{4}$	$3.0614\times 10^{3}$	$2.6063\times 10^{2}$
BA	$5.3308\times 10^{3}$	$2.8462\times 10^{3}$	$1.3142\times 10^{5}$	$8.8291\times 10^{4}$	$3.5548\times 10^{3}$	$5.8589\times 10^{2}$
MGO	$1.4594\times 10^{4}$	$1.0484\times 10^{4}$	$1.9265\times 10^{4}$	$1.7824\times 10^{4}$	$2.2420\times 10^{3}$	$1.2348\times 10^{2}$
MFO	$2.0815\times 10^{5}$	$3.7231\times 10^{5}$	$5.7746\times 10^{4}$	$4.6224\times 10^{4}$	$3.1174\times 10^{3}$	$3.9176\times 10^{2}$
FA	$1.9457\times 10^{5}$	$8.7780\times 10^{4}$	$7.0943\times 10^{7}$	$2.3455\times 10^{7}$	$3.4622\times 10^{3}$	$1.5254\times 10^{2}$
HGS	$4.5232\times 10^{4}$	$3.6473\times 10^{4}$	$1.8705\times 10^{4}$	$1.3263\times 10^{4}$	$2.6707\times 10^{3}$	$3.6892\times 10^{2}$
GWO	$2.0773\times 10^{5}$	$3.0614\times 10^{5}$	$1.4027\times 10^{6}$	$6.2694\times 10^{6}$	$2.3334\times 10^{3}$	$2.0441\times 10^{2}$
SMA	$1.9318\times 10^{5}$	$9.9722\times 10^{4}$	$1.9264\times 10^{4}$	$8.2019\times 10^{3}$	$2.8121\times 10^{3}$	$2.3952\times 10^{2}$
SCA	$1.2616\times 10^{5}$	$1.0908\times 10^{5}$	$1.9482\times 10^{7}$	$1.6034\times 10^{7}$	$3.6082\times 10^{3}$	$1.7743\times 10^{2}$
	**F17**		**F18**		**F19**	
**Algo.**	**Avg**	**Std**	**Avg**	**Std**	**Avg**	**Std**
CL-PO	$1.8478\times 10^{3}$	$9.8444\times 10^{1}$	$1.8435\times 10^{3}$	$1.3147\times 10^{1}$	$1.9142\times 10^{3}$	$4.8961\times 10^{0}$
PO	$2.2342\times 10^{3}$	$2.1217\times 10^{2}$	$4.8090\times 10^{5}$	$3.8444\times 10^{5}$	$7.4146\times 10^{5}$	$5.7147\times 10^{5}$
BA	$2.7559\times 10^{3}$	$3.5513\times 10^{2}$	$1.7425\times 10^{5}$	$1.1412\times 10^{5}$	$6.3575\times 10^{5}$	$2.7391\times 10^{5}$
MGO	$1.8771\times 10^{3}$	$5.3466\times 10^{1}$	$4.1539\times 10^{5}$	$3.0121\times 10^{5}$	$1.8287\times 10^{4}$	$2.8500\times 10^{4}$
MFO	$2.4886\times 10^{3}$	$2.7744\times 10^{2}$	$2.3606\times 10^{6}$	$5.6712\times 10^{6}$	$1.6429\times 10^{7}$	$4.4743\times 10^{7}$
FA	$2.4835\times 10^{3}$	$1.2428\times 10^{2}$	$4.1353\times 10^{6}$	$1.6014\times 10^{6}$	$1.0457\times 10^{8}$	$4.2858\times 10^{7}$
HGS	$2.2386\times 10^{3}$	$2.1062\times 10^{2}$	$3.2062\times 10^{5}$	$3.0939\times 10^{5}$	$2.1406\times 10^{4}$	$1.9712\times 10^{4}$
GWO	$1.9642\times 10^{3}$	$1.1726\times 10^{2}$	$8.8107\times 10^{5}$	$9.5800\times 10^{5}$	$4.3149\times 10^{5}$	$4.1825\times 10^{5}$
SMA	$2.2843\times 10^{3}$	$2.2101\times 10^{2}$	$6.6021\times 10^{5}$	$5.7411\times 10^{5}$	$3.2449\times 10^{5}$	$3.2562\times 10^{5}$
SCA	$2.3544\times 10^{3}$	$1.4140\times 10^{2}$	$2.7822\times 10^{6}$	$1.5082\times 10^{6}$	$2.5087\times 10^{7}$	$1.0861\times 10^{7}$
	**F20**		**F21**		**F22**	
**Algo.**	**Avg**	**Std**	**Avg**	**Std**	**Avg**	**Std**
CL-PO	$2.2357\times 10^{3}$	$9.9106\times 10^{1}$	$2.3642\times 10^{3}$	$1.4610\times 10^{1}$	$3.0999\times 10^{3}$	$1.1593\times 10^{3}$
PO	$2.5754\times 10^{3}$	$1.7053\times 10^{2}$	$2.4978\times 10^{3}$	$7.2631\times 10^{1}$	$3.1000\times 10^{3}$	$1.5281\times 10^{3}$
BA	$2.9587\times 10^{3}$	$2.0478\times 10^{2}$	$2.6255\times 10^{3}$	$7.6594\times 10^{1}$	$7.2673\times 10^{3}$	$1.3390\times 10^{3}$
MGO	$2.2219\times 10^{3}$	$8.0486\times 10^{1}$	$2.3576\times 10^{3}$	$3.3524\times 10^{1}$	$3.4777\times 10^{3}$	$1.8210\times 10^{3}$
MFO	$2.7774\times 10^{3}$	$2.3974\times 10^{2}$	$2.4914\times 10^{3}$	$4.8186\times 10^{1}$	$6.5165\times 10^{3}$	$1.3358\times 10^{3}$
FA	$2.5996\times 10^{3}$	$9.6909\times 10^{1}$	$2.5385\times 10^{3}$	$1.2648\times 10^{1}$	$3.8328\times 10^{3}$	$1.5490\times 10^{2}$
HGS	$2.5190\times 10^{3}$	$1.6432\times 10^{2}$	$2.4280\times 10^{3}$	$3.0547\times 10^{1}$	$4.9520\times 10^{3}$	$1.5790\times 10^{3}$
GWO	$2.3594\times 10^{3}$	$1.1742\times 10^{2}$	$2.3837\times 10^{3}$	$2.0381\times 10^{1}$	$4.7471\times 10^{3}$	$1.4303\times 10^{3}$
SMA	$2.4295\times 10^{3}$	$1.2974\times 10^{2}$	$2.4798\times 10^{3}$	$3.0765\times 10^{1}$	$4.4077\times 10^{3}$	$2.3263\times 10^{3}$
SCA	$2.6171\times 10^{3}$	$1.2920\times 10^{2}$	$2.5516\times 10^{3}$	$1.9900\times 10^{1}$	$8.8117\times 10^{3}$	$1.9180\times 10^{3}$
	**F23**		**F24**		**F25**	
**Algo.**	**Avg**	**Std**	**Avg**	**Std**	**Avg**	**Std**
CL-PO	$2.7164\times 10^{3}$	$1.5075\times 10^{1}$	$2.9435\times 10^{3}$	$3.3948\times 10^{1}$	$2.8863\times 10^{3}$	$1.6677\times 10^{0}$
PO	$2.9631\times 10^{3}$	$8.3546\times 10^{1}$	$3.1020\times 10^{3}$	$6.4613\times 10^{1}$	$2.9367\times 10^{3}$	$3.0938\times 10^{1}$
BA	$3.3185\times 10^{3}$	$1.3625\times 10^{2}$	$3.3751\times 10^{3}$	$1.3323\times 10^{2}$	$2.9126\times 10^{3}$	$2.5532\times 10^{1}$
MGO	$2.7179\times 10^{3}$	$1.3239\times 10^{1}$	$2.8964\times 10^{3}$	$1.3368\times 10^{1}$	$2.8873\times 10^{3}$	$7.2690\times 10^{-1}$
MFO	$2.8463\times 10^{3}$	$4.1009\times 10^{1}$	$2.9962\times 10^{3}$	$3.0696\times 10^{1}$	$3.3408\times 10^{3}$	$5.7788\times 10^{2}$
FA	$2.9107\times 10^{3}$	$1.6163\times 10^{1}$	$3.0632\times 10^{3}$	$1.2673\times 10^{1}$	$3.5721\times 10^{3}$	$7.2093\times 10^{1}$
HGS	$2.7727\times 10^{3}$	$3.0516\times 10^{1}$	$3.0173\times 10^{3}$	$6.0892\times 10^{1}$	$2.8921\times 10^{3}$	$1.3669\times 10^{1}$
GWO	$2.7538\times 10^{3}$	$2.9431\times 10^{1}$	$2.9110\times 10^{3}$	$4.3349\times 10^{1}$	$2.9801\times 10^{3}$	$3.2877\times 10^{1}$
SMA	$2.8569\times 10^{3}$	$2.7354\times 10^{1}$	$3.0093\times 10^{3}$	$3.5180\times 10^{1}$	$3.0253\times 10^{3}$	$4.4992\times 10^{1}$
SCA	$2.9887\times 10^{3}$	$2.3861\times 10^{1}$	$3.1605\times 10^{3}$	$3.0713\times 10^{1}$	$3.2009\times 10^{3}$	$6.3095\times 10^{1}$
	**F26**		**F27**		**F28**	
**Algo.**	**Avg**	**Std**	**Avg**	**Std**	**Avg**	**Std**
CL-PO	$3.8840\times 10^{3}$	$7.9212\times 10^{2}$	$3.2071\times 10^{3}$	$1.4430\times 10^{1}$	$3.1325\times 10^{3}$	$5.5804\times 10^{1}$
PO	$6.4550\times 10^{3}$	$1.6443\times 10^{3}$	$3.3031\times 10^{3}$	$3.8488\times 10^{1}$	$3.2975\times 10^{3}$	$3.3813\times 10^{1}$
BA	$9.1851\times 10^{3}$	$2.0334\times 10^{3}$	$3.4434\times 10^{3}$	$1.5417\times 10^{2}$	$3.1296\times 10^{3}$	$5.6033\times 10^{1}$
MGO	$4.0487\times 10^{3}$	$4.8354\times 10^{2}$	$3.2119\times 10^{3}$	$7.0889\times 10^{0}$	$3.2316\times 10^{3}$	$1.4316\times 10^{1}$
MFO	$6.0526\times 10^{3}$	$4.3190\times 10^{2}$	$3.2673\times 10^{3}$	$2.8908\times 10^{1}$	$4.1014\times 10^{3}$	$8.0540\times 10^{2}$
FA	$6.5179\times 10^{3}$	$1.5986\times 10^{2}$	$3.3301\times 10^{3}$	$2.1701\times 10^{1}$	$3.9020\times 10^{3}$	$1.0075\times 10^{2}$
HGS	$4.8561\times 10^{3}$	$6.1851\times 10^{2}$	$3.2257\times 10^{3}$	$1.3370\times 10^{1}$	$3.2053\times 10^{3}$	$5.4102\times 10^{1}$
GWO	$4.5295\times 10^{3}$	$2.8691\times 10^{2}$	$3.2458\times 10^{3}$	$2.7535\times 10^{1}$	$3.4028\times 10^{3}$	$6.6148\times 10^{1}$
SMA	$5.1274\times 10^{3}$	$7.7707\times 10^{2}$	$3.2543\times 10^{3}$	$2.7627\times 10^{1}$	$3.4127\times 10^{3}$	$4.2872\times 10^{1}$
SCA	$6.9248\times 10^{3}$	$3.0357\times 10^{2}$	$3.3961\times 10^{3}$	$4.1810\times 10^{1}$	$3.8534\times 10^{3}$	$1.6350\times 10^{2}$
	**F29**		**F30**			
**Algo.**	**Avg**	**Std**	**Avg**	**Std**		
CL-PO	$3.4633\times 10^{3}$	$1.1614\times 10^{2}$	$5.1453\times 10^{3}$	$1.9215\times 10^{2}$		
PO	$4.5677\times 10^{3}$	$3.1516\times 10^{2}$	$6.8410\times 10^{6}$	$5.2652\times 10^{6}$		
BA	$4.9524\times 10^{3}$	$4.8279\times 10^{2}$	$1.3912\times 10^{6}$	$9.3791\times 10^{5}$		
MGO	$3.6368\times 10^{3}$	$7.4555\times 10^{1}$	$7.9722\times 10^{4}$	$6.3679\times 10^{4}$		
MFO	$4.1171\times 10^{3}$	$2.7124\times 10^{2}$	$1.0023\times 10^{6}$	$1.6390\times 10^{6}$		
FA	$4.6674\times 10^{3}$	$1.6825\times 10^{2}$	$9.2213\times 10^{7}$	$3.1391\times 10^{7}$		
HGS	$3.7634\times 10^{3}$	$1.6171\times 10^{2}$	$6.5111\times 10^{4}$	$1.0480\times 10^{5}$		
GWO	$3.7846\times 10^{3}$	$1.4906\times 10^{2}$	$6.6650\times 10^{6}$	$6.4928\times 10^{6}$		
SMA	$3.9622\times 10^{3}$	$2.4947\times 10^{2}$	$5.2040\times 10^{6}$	$3.2022\times 10^{6}$		
SCA	$4.5392\times 10^{3}$	$1.4055\times 10^{2}$	$7.5290\times 10^{7}$	$2.6456\times 10^{7}$		
**Overall Rank**						
**Algo.**	**RANK**	**+/=/−**	**AVG**			
CL-PO	1		1.2759			
PO	6	27/2/0	5.8276			
BA	7	27/2/0	6.931			
MGO	2	17/10/2	2.5517			
MFO	8	29/0/0	7.3103			
FA	9	29/0/0	8.4828			
HGS	3	28/1/0	3.5172			
GWO	4	28/0/1	4.8276			
SMA	5	29/0/0	5.7931			
SCA	9	29/0/0	8.4828			

**Table 3:** The *p*-values of the CL-PO versus other algorithms on CEC2017.

Fun	PO	BA	MGO	MFO	FA	HGS	GWO	SMA	SCA
F1	$1.73\times 10^{-6}$	$1.73\times 10^{-6}$	$1.73\times 10^{-6}$	$1.73\times 10^{-6}$	$1.73\times 10^{-6}$	$1.73\times 10^{-6}$	$1.73\times 10^{-6}$	$1.73\times 10^{-6}$	$1.73\times 10^{-6}$
F3	$1.73\times 10^{-6}$	$7.34\times 10^{-1}$	$1.73\times 10^{-6}$	$1.73\times 10^{-6}$	$1.73\times 10^{-6}$	$1.73\times 10^{-6}$	$1.73\times 10^{-6}$	$1.73\times 10^{-6}$	$1.73\times 10^{-6}$
F4	$1.73\times 10^{-6}$	$2.35\times 10^{-6}$	$1.73\times 10^{-6}$	$1.73\times 10^{-6}$	$1.73\times 10^{-6}$	$5.22\times 10^{-6}$	$1.73\times 10^{-6}$	$1.73\times 10^{-6}$	$1.73\times 10^{-6}$
F5	$1.73\times 10^{-6}$	$1.73\times 10^{-6}$	$2.85\times 10^{-2}$	$1.73\times 10^{-6}$	$1.73\times 10^{-6}$	$1.73\times 10^{-6}$	$2.13\times 10^{-6}$	$1.73\times 10^{-6}$	$1.73\times 10^{-6}$
F6	$1.73\times 10^{-6}$	$1.73\times 10^{-6}$	$1.06\times 10^{-1}$	$1.73\times 10^{-6}$	$1.73\times 10^{-6}$	$1.73\times 10^{-6}$	$1.73\times 10^{-6}$	$1.73\times 10^{-6}$	$1.73\times 10^{-6}$
F7	$1.73\times 10^{-6}$	$1.73\times 10^{-6}$	$1.06\times 10^{-1}$	$1.73\times 10^{-6}$	$1.73\times 10^{-6}$	$1.73\times 10^{-6}$	$1.73\times 10^{-6}$	$1.73\times 10^{-6}$	$1.73\times 10^{-6}$
F8	$1.73\times 10^{-6}$	$1.73\times 10^{-6}$	$4.41\times 10^{-1}$	$1.73\times 10^{-6}$	$1.73\times 10^{-6}$	$1.73\times 10^{-6}$	$3.52\times 10^{-6}$	$1.73\times 10^{-6}$	$1.73\times 10^{-6}$
F9	$1.73\times 10^{-6}$	$1.73\times 10^{-6}$	$4.78\times 10^{-1}$	$1.73\times 10^{-6}$	$1.73\times 10^{-6}$	$1.73\times 10^{-6}$	$4.53\times 10^{-6}$	$1.73\times 10^{-6}$	$1.73\times 10^{-6}$
F10	$1.73\times 10^{-6}$	$1.73\times 10^{-6}$	$3.38\times 10^{-3}$	$1.73\times 10^{-6}$	$1.73\times 10^{-6}$	$1.73\times 10^{-6}$	$1.73\times 10^{-6}$	$1.73\times 10^{-6}$	$1.73\times 10^{-6}$
F11	$1.73\times 10^{-6}$	$1.73\times 10^{-6}$	$1.92\times 10^{-6}$	$1.73\times 10^{-6}$	$1.73\times 10^{-6}$	$2.60\times 10^{-6}$	$9.32\times 10^{-6}$	$1.73\times 10^{-6}$	$1.73\times 10^{-6}$
F12	$1.73\times 10^{-6}$	$1.73\times 10^{-6}$	$1.73\times 10^{-6}$	$1.73\times 10^{-6}$	$1.73\times 10^{-6}$	$1.73\times 10^{-6}$	$1.73\times 10^{-6}$	$1.73\times 10^{-6}$	$1.73\times 10^{-6}$
F13	$1.73\times 10^{-6}$	$1.73\times 10^{-6}$	$1.73\times 10^{-6}$	$1.73\times 10^{-6}$	$1.73\times 10^{-6}$	$1.73\times 10^{-6}$	$1.73\times 10^{-6}$	$1.73\times 10^{-6}$	$1.73\times 10^{-6}$
F14	$1.73\times 10^{-6}$	$1.73\times 10^{-6}$	$1.73\times 10^{-6}$	$1.73\times 10^{-6}$	$1.73\times 10^{-6}$	$1.73\times 10^{-6}$	$1.73\times 10^{-6}$	$1.73\times 10^{-6}$	$1.73\times 10^{-6}$
F15	$1.73\times 10^{-6}$	$1.73\times 10^{-6}$	$1.73\times 10^{-6}$	$1.73\times 10^{-6}$	$1.73\times 10^{-6}$	$1.73\times 10^{-6}$	$1.73\times 10^{-6}$	$1.73\times 10^{-6}$	$1.73\times 10^{-6}$
F16	$1.73\times 10^{-6}$	$1.73\times 10^{-6}$	$2.90\times 10^{-1}$	$1.73\times 10^{-6}$	$1.73\times 10^{-6}$	$1.36\times 10^{-5}$	$1.48\times 10^{-2}$	$1.73\times 10^{-6}$	$1.73\times 10^{-6}$
F17	$3.18\times 10^{-6}$	$2.13\times 10^{-6}$	$1.47\times 10^{-1}$	$1.73\times 10^{-6}$	$1.73\times 10^{-6}$	$1.73\times 10^{-6}$	$1.89\times 10^{-4}$	$2.13\times 10^{-6}$	$1.73\times 10^{-6}$
F18	$1.73\times 10^{-6}$	$1.73\times 10^{-6}$	$1.73\times 10^{-6}$	$1.73\times 10^{-6}$	$1.73\times 10^{-6}$	$1.73\times 10^{-6}$	$1.73\times 10^{-6}$	$1.73\times 10^{-6}$	$1.73\times 10^{-6}$
F19	$1.73\times 10^{-6}$	$1.73\times 10^{-6}$	$1.73\times 10^{-6}$	$1.73\times 10^{-6}$	$1.73\times 10^{-6}$	$1.73\times 10^{-6}$	$1.73\times 10^{-6}$	$1.73\times 10^{-6}$	$1.73\times 10^{-6}$
F20	$4.29\times 10^{-6}$	$1.73\times 10^{-6}$	$6.73\times 10^{-1}$	$1.73\times 10^{-6}$	$1.92\times 10^{-6}$	$4.29\times 10^{-6}$	$1.36\times 10^{-4}$	$3.72\times 10^{-5}$	$1.73\times 10^{-6}$
F21	$6.98\times 10^{-6}$	$1.73\times 10^{-6}$	$9.59\times 10^{-1}$	$1.73\times 10^{-6}$	$1.73\times 10^{-6}$	$2.35\times 10^{-6}$	$1.36\times 10^{-4}$	$1.73\times 10^{-6}$	$1.73\times 10^{-6}$
F22	$3.49\times 10^{-1}$	$2.88\times 10^{-6}$	$4.28\times 10^{-2}$	$2.13\times 10^{-6}$	$2.61\times 10^{-6}$	$4.86\times 10^{-5}$	$9.71\times 10^{-5}$	$4.11\times 10^{-3}$	$1.73\times 10^{-6}$
F23	$1.73\times 10^{-6}$	$1.73\times 10^{-6}$	$6.44\times 10^{-1}$	$1.73\times 10^{-6}$	$1.73\times 10^{-6}$	$1.73\times 10^{-6}$	$3.52\times 10^{-6}$	$1.73\times 10^{-6}$	$1.73\times 10^{-6}$
F24	$1.73\times 10^{-6}$	$1.73\times 10^{-6}$	$4.73\times 10^{-6}$	$1.24\times 10^{-5}$	$1.73\times 10^{-6}$	$1.24\times 10^{-5}$	$1.38\times 10^{-3}$	$6.98\times 10^{-6}$	$1.73\times 10^{-6}$
F25	$2.35\times 10^{-6}$	$3.18\times 10^{-6}$	$1.97\times 10^{-5}$	$1.73\times 10^{-6}$	$1.73\times 10^{-6}$	$1.25\times 10^{-1}$	$1.73\times 10^{-6}$	$1.73\times 10^{-6}$	$1.73\times 10^{-6}$
F26	$1.92\times 10^{-6}$	$2.13\times 10^{-6}$	$5.30\times 10^{-1}$	$1.73\times 10^{-6}$	$1.73\times 10^{-6}$	$2.84\times 10^{-5}$	$3.61\times 10^{-3}$	$1.80\times 10^{-5}$	$1.73\times 10^{-6}$
F27	$1.73\times 10^{-6}$	$1.73\times 10^{-6}$	$2.37\times 10^{-1}$	$1.73\times 10^{-6}$	$1.73\times 10^{-6}$	$5.79\times 10^{-5}$	$4.73\times 10^{-6}$	$1.73\times 10^{-6}$	$1.73\times 10^{-6}$
F28	$2.13\times 10^{-6}$	$1.71\times 10^{-1}$	$4.73\times 10^{-6}$	$1.73\times 10^{-6}$	$1.73\times 10^{-6}$	$1.61\times 10^{-4}$	$1.73\times 10^{-6}$	$1.73\times 10^{-6}$	$1.73\times 10^{-6}$
F29	$1.73\times 10^{-6}$	$1.73\times 10^{-6}$	$3.41\times 10^{-5}$	$1.73\times 10^{-6}$	$1.73\times 10^{-6}$	$6.34\times 10^{-6}$	$3.52\times 10^{-6}$	$1.92\times 10^{-6}$	$1.73\times 10^{-6}$
F30	$1.73\times 10^{-6}$	$1.73\times 10^{-6}$	$1.73\times 10^{-6}$	$1.73\times 10^{-6}$	$1.73\times 10^{-6}$	$1.73\times 10^{-6}$	$1.73\times 10^{-6}$	$1.73\times 10^{-6}$	$1.73\times 10^{-6}$

**Table 4:** Comparative results for the reservoir production optimization problem.

Algorithm	Mean NPV (USD)	Std	Best	Worst
CL-PO	$9.625\times 10^{8}$	$1.250\times 10^{7}$	$9.810\times 10^{8}$	$9.445\times 10^{8}$
PO	$8.755\times 10^{8}$	$3.224\times 10^{7}$	$9.121\times 10^{8}$	$8.401\times 10^{8}$
BA	$7.936\times 10^{8}$	$4.721\times 10^{7}$	$8.450\times 10^{8}$	$7.426\times 10^{8}$
MGO	$9.435\times 10^{8}$	$1.892\times 10^{7}$	$9.688\times 10^{8}$	$9.183\times 10^{8}$
MFO	$8.272\times 10^{8}$	$4.113\times 10^{7}$	$8.705\times 10^{8}$	$7.841\times 10^{8}$
FA	$8.104\times 10^{8}$	$4.398\times 10^{7}$	$8.595\times 10^{8}$	$7.614\times 10^{8}$
HGS	$9.282\times 10^{8}$	$2.147\times 10^{7}$	$9.578\times 10^{8}$	$8.994\times 10^{8}$
GWO	$9.108\times 10^{8}$	$2.485\times 10^{7}$	$9.421\times 10^{8}$	$8.805\times 10^{8}$
SMA	$8.961\times 10^{8}$	$2.830\times 10^{7}$	$9.302\times 10^{8}$	$8.637\times 10^{8}$
SCA	$8.541\times 10^{8}$	$3.665\times 10^{7}$	$8.931\times 10^{8}$	$8.162\times 10^{8}$

## Data Availability

The code and data are available from the corresponding author on reasonable request.

## References

[1] Rao S.S. (2019). Engineering Optimization: Theory and Practice.

[2] Belegundu A.D., Chandrupatla T.R. (2019). Optimization Concepts and Applications in Engineering.

[3] Du J., Hu H., Wang J. A Two-Stage Multi-Evolutionary Sampling Optimization Framework for Expensive Optimization Problems. Proceedings of the 2025 International Conference on New Trends in Computational Intelligence (NTCI).

[4] Hu H., Shan W., Tang Y., Heidari A.A., Chen H., Liu H., Wang M., Escorcia-Gutierrez J., Mansour R.F., Chen J. (2022). Horizontal and vertical crossover of sine cosine algorithm with quick moves for optimization and feature selection. J. Comput. Des. Eng..

[5] Shan W., Hu H., Cai Z., Chen H., Liu H., Wang M., Teng Y. (2022). Multi-strategies boosted mutative crow search algorithm for global tasks: Cases of continuous and discrete optimization. J. Bionic Eng..

[6] Abualigah L., Elaziz M.A., Khasawneh A.M., Alshinwan M., Ibrahim R.A., Al-Qaness M.A., Mirjalili S., Sumari P., Gandomi A.H. (2022). Meta-heuristic optimization algorithms for solving real-world mechanical engineering design problems: A comprehensive survey, applications, comparative analysis, and results. Neural Comput. Appl..

[7] Hu H., Wang J., Huang X., Ablameyko S.V. An Integrated Online-Offline Hybrid Particle Swarm Optimization Framework for Medium Scale Expensive Problems. Proceedings of the 2024 6th International Conference on Data-driven Optimization of Complex Systems (DOCS).

[8] Kar B., Yahya W., Lin Y.D., Ali A. (2023). Offloading using traditional optimization and machine learning in federated cloud–edge–fog systems: A survey. IEEE Commun. Surv. Tutorials.

[9] Zeng J., Yin W. (2018). On nonconvex decentralized gradient descent. IEEE Trans. Signal Process..

[10] Kiwiel K.C. (2006). Methods of Descent for Nondifferentiable Optimization.

[11] Shao S., Tian Y., Zhang Y., Zhang X. (2025). Knowledge learning-based dimensionality reduction for solving large-scale sparse multiobjective optimization problems. IEEE Trans. Cybern..

[12] Hu H., Shan W., Chen J., Xing L., Heidari A.A., Chen H., He X., Wang M. (2023). Dynamic individual selection and crossover boosted forensic-based investigation algorithm for global optimization and feature selection. J. Bionic Eng..

[13] Boussaïd I., Lepagnot J., Siarry P. (2013). A survey on optimization metaheuristics. Inf. Sci..

[14] Feyel P. (2017). Robust Control Optimization with Metaheuristics.

[15] Tu J., Chen H., Wang M., Gandomi A.H. (2021). The colony predation algorithm. J. Bionic Eng..

[16] Zheng B., Chen Y., Wang C., Heidari A.A., Liu L., Chen H. (2024). The moss growth optimization (MGO): Concepts and performance. J. Comput. Des. Eng..

[17] Lian J., Zhu T., Ma L., Wu X., Heidari A.A., Chen Y., Chen H., Hui G. (2024). The educational competition optimizer. Int. J. Syst. Sci..

[18] Ouyang K., Wei D., Sha X., Yu J., Zhao Y., Qiu M., Fu S., Heidari A.A., Chen H. (2025). Beaver behavior optimizer: A novel metaheuristic algorithm for solar PV parameter identification and engineering problems. J. Adv. Res..

[19] Lian J., Hui G., Ma L., Zhu T., Wu X., Heidari A.A., Chen Y., Chen H. (2024). Parrot optimizer: Algorithm and applications to medical problems. Comput. Biol. Med..

[20] Cao S., Li W., Huang K., Deng X., Li R. (2025). Optimization of EHA Hydraulic Cylinder Buffer Design Using Enhanced SBO–BP Neural Network and NSGA-II. Mathematics.

[21] Ji Y., Cai Z., Yin Z. (2026). Enhancing Status-Based Optimization via Dual Dispersal Mechanisms for Feature Selection in Bankruptcy Prediction. Neurocomputing.

[22] Hussain K., Salleh M.N.M., Cheng S., Shi Y. (2019). On the exploration and exploitation in popular swarm-based metaheuristic algorithms. Neural Comput. Appl..

[23] Sastry K., Goldberg D.E., Kendall G. (2013). Genetic algorithms. Search Methodologies: Introductory Tutorials in Optimization and Decision Support Techniques.

[24] Storn R., Price K. (1997). Differential evolution—A simple and efficient heuristic for global optimization over continuous spaces. J. Glob. Optim..

[25] Vanneschi L., Silva S. (2023). Particle swarm optimization. Lectures on Intelligent Systems.

[26] Dorigo M., Birattari M., Stutzle T. (2007). Ant colony optimization. IEEE Comput. Intell. Mag..

[27] Wolpert D.H., Macready W.G. (2002). No free lunch theorems for optimization. IEEE Trans. Evol. Comput..

[28] Liang J.J., Qin A.K., Suganthan P.N., Baskar S. (2006). Comprehensive learning particle swarm optimizer for global optimization of multimodal functions. IEEE Trans. Evol. Comput..

[29] Yang Y., Chen H., Heidari A.A., Gandomi A.H. (2021). Hunger games search: Visions, conception, implementation, deep analysis, perspectives, and towards performance shifts. Expert Syst. Appl..

[30] Mirjalili S., Mirjalili S.M., Lewis A. (2014). Grey wolf optimizer. Adv. Eng. Softw..

[31] Li S., Chen H., Wang M., Heidari A.A., Mirjalili S. (2020). Slime mould algorithm: A new method for stochastic optimization. Future Gener. Comput. Syst..

[32] Mirjalili S. (2016). SCA: A sine cosine algorithm for solving optimization problems. Knowl.-Based Syst..

[33] Yang X.S., Slowik A. (2020). Firefly algorithm. Swarm Intelligence Algorithms.

[34] Yang X.S. (2011). Bat algorithm for multi-objective optimisation. Int. J. Bio-Inspired Comput..

[35] Ren X., Wang H., Hu H., Wang J., Ablameyko S.V. (2025). Weighted committee-based surrogate-assisted differential evolution framework for efficient medium-scale expensive optimization. Int. J. Mach. Learn. Cybern..

[36] Jansen J.D., Fonseca R.M., Kahrobaei S., Siraj M., Van Essen G., Van den Hof P. (2014). The egg model–a geological ensemble for reservoir simulation. Geosci. Data J..

[37] Giannelos S., Konstantelos I., Pudjianto D., Strbac G. (2026). The impact of electrolyser allocation on Great Britain’s electricity transmission system in 2050. Int. J. Hydrogen Energy.

[38] Kaloev M., Krastev G. Tailored Learning Rates for Reinforcement Learning: A Visual Exploration and Guideline Formulation. Proceedings of the 2023 7th International Symposium on Innovative Approaches in Smart Technologies (ISAS).

